# Supplementary material for: Bayesian Meta-Analysis of Myopia Control with Multifocal Lenses
Source: J Clin Med. 2021 Feb 12;10(4):730. doi: 10.3390/jcm10040730 (PMC7917905; doi:10.3390/jcm10040730)
Supplement: Supplementary file 1 [file jcm-10-00730-s001.pdf]

Supplementary Electronic Material

Table S1: Scaled progressions in SER and their standard errors in the selected trials for the 6, 12, 18, 24 and 36-months follow-ups from baseline. (NC NT) – number of subjects who completed the trial in the control and treatment groups, respectively; N/A – not available.

Trial	Addition (D) (NC NT)	Change in SER at 6M (SE)	Change in SER at 12M (SE)	Change in SER at 18M (SE)	Change in SER at 24M (SE)	Change in SER at 36M (SE)
Leung (1999)	+1.50 (32C 22T)	0.742 (0.324)	0.656 (0.153)	0.707 (0.108)	0.618 (0.079)	N/A
Edwards (2002)	+1.50 (133C 121T)	0.674 (0.157)	0.753 (0.103)	0.806 (0.083)	0.868 (0.066)	N/A
Gwiazda (2003)	+2.00 (234C 235T)	N/A	0.700 (0.061)	N/A	0.813 (0.048)	0.865 (0.054)
Hao (2006)	N/A (87C 95T)	0.718 (0.276)	0.740 (0.149)	0.864 (0.114)	0.931 (0.094)	0.912 (0.063)
Hasebe (2008)	+1.50 (44C 46T)	0.592 (0.155)	0.612 (0.099)	0.720 (0.106)	N/A	N/A
Yang (2009)	+1.50 (72C 73T)	0.944 (0.251)	0.831 (0.114)	0.845 (0.081)	0.827 (0.064)	N/A
COMET2 Group (2011)	+2.00 (58C 56T)	N/A	0.690 (0.145)	N/A	0.725 (0.114)	0.757 (0.109)
Shih (2011)	N/A (76C 75T)	0.833 (0.142)	0.860 (0.098)	0.850 (0.074)	N/A	N/A
Berntsen (2012)	+2.00 (43C 41T)	N/A	0.673 (0.124)	N/A	N/A	N/A
Hasebe (2014)	+1.50 (63C 56T)	0.804 (0.137)	0.700 (0.082)	0.800 (0.080)	0.806 (0.073)	N/A

Table S2: Scaled progressions in AL and their standard errors in the selected trials for 6, 12, 18 and 24-months intervals from baseline. (NC NT) – number of subjects who completed the trial in the control and treatment groups, respectively; N/A – not available.

Trial	Addition (D) (NC NT)	Change in AL at 6M at (SE)	Change in AL at 12M (SE)	Change in AL at 18M (SE)	Change in AL at 24M (SE)
Leung & Brown (1999)	+1.50 (32C 22T)	0.714 (0.258)	0.685 (0.159)	0.691 (0.108)	0.660 (0.092)
Edwards et al. (2002)	+1.50 (133C 121T)	0.932 (0.125)	0.973 (0.086)	0.967 (0.063)	0.975 (0.051)
Gwiazda et al. (2003)	+2.00 (234C 235T)	N/A	0.804 (0.054)	N/A	0.850 (0.032)
Hao et al. (2006)	N/A (87C 95T)	N/A	N/A	N/A	N/A
Hasebe et al. (2008)	+1.50 (44C 46T)	N/A	N/A	N/A	N/A
Yang et al. (2009)	+1.50 (72C 73T)	0.810 (0.213)	0.667 (0.097)	0.945 (0.091)	0.855 (0.070)
COMET2 (2011)	+2.00 (58C 56T)	N/A	N/A	N/A	N/A
Shih et al. (2011)	N/A (76C 75T)	N/A	N/A	0.831 (0.076)	N/A
Berntsen et al. (2012)	+2.00 (43C 41T)	N/A	0.793 (0.114)	N/A	N/A
Hasebe et al. (2014)	+1.50 (63C 56T)	0.888 (0.075)	0.868 (0.061)	0.916 (0.061)	0.880 (0.061)

Table S3: The sensitivity analysis for the scaled progression in SER, including all the trials and excluding the 4 equivocal trials, at the 12-months follow-up.

	All the trials were included		The 4 equivocal trials were excluded	
Prior	μ (SE)	τ(SE)	μ (SE)	τ (SE)
$\mu \sim N(0.7, 0.1^2)$ $\tau \sim$ HC(0, 0.1)	0.719 (0.033)	0.037 (0.030)	0.699 (0.069)	0.042 (0.037)
$\mu \sim N(0.6, 0.1^2)$ $\tau \sim$ HC(0, 0.1)	0.708 (0.034)	0.037 (0.030)	0.683 (0.041)	0.043 (0.038)
$\mu \sim N(0.8, 0.1^2)$ $\tau \sim$ HC(0, 0.1)	0.731 (0.034)	0.037 (0.030)	0.716 (0.041)	0.043 (0.038)
$\mu \sim N(0.6, 0.2^2)$ $\tau \sim$ HC(0, 0.1)	0.718 (0.035)	0.037 (0.030)	0.694 (0.044)	0.043 (0.038)
$\mu \sim N(0.8, 0.2^2)$ $\tau \sim$ HC(0, 0.1)	0.724 (0.035)	0.037 (0.030)	0.703 (0.044)	0.043 (0.038)
$\mu \sim N(0.6, 0.1^2)$ $\tau \sim$ HC(0, 0.2)	0.707 (0.035)	0.042 (0.034)	0.681 (0.042)	0.052 (0.046)
$\mu \sim N(0.8, 0.1^2)$ $\tau \sim$ HC(0, 0.2)	0.731 (0.035)	0.042 (0.034)	0.717 (0.043)	0.052 (0.046)
$\mu \sim N(0.7, 0.1^2)$ $\tau \sim$ HC(0, 0.2)	0.719 (0.034)	0.042 (0.034)	0.699 (0.042)	0.050 (0.044)
$\mu \sim N(0.7, 0.1^2)$ $\tau \sim$ HC(0, 0.3)	0.719 (0.035)	0.043 (0.035)	0.699 (0.043)	0.053 (0.048)
$\mu \sim N(0.7, 0.2^2)$ $\tau \sim$ HC(0, 0.2)	0.721 (0.036)	0.042 (0.034)	0.699 (0.046)	0.052 (0.046)
$\mu \sim N(0.7, 0.2^2)$ $\tau \sim$ HC(0, 0.3)	0.721 (0.037)	0.044 (0.036)	0.699 (0.047)	0.055 (0.050)

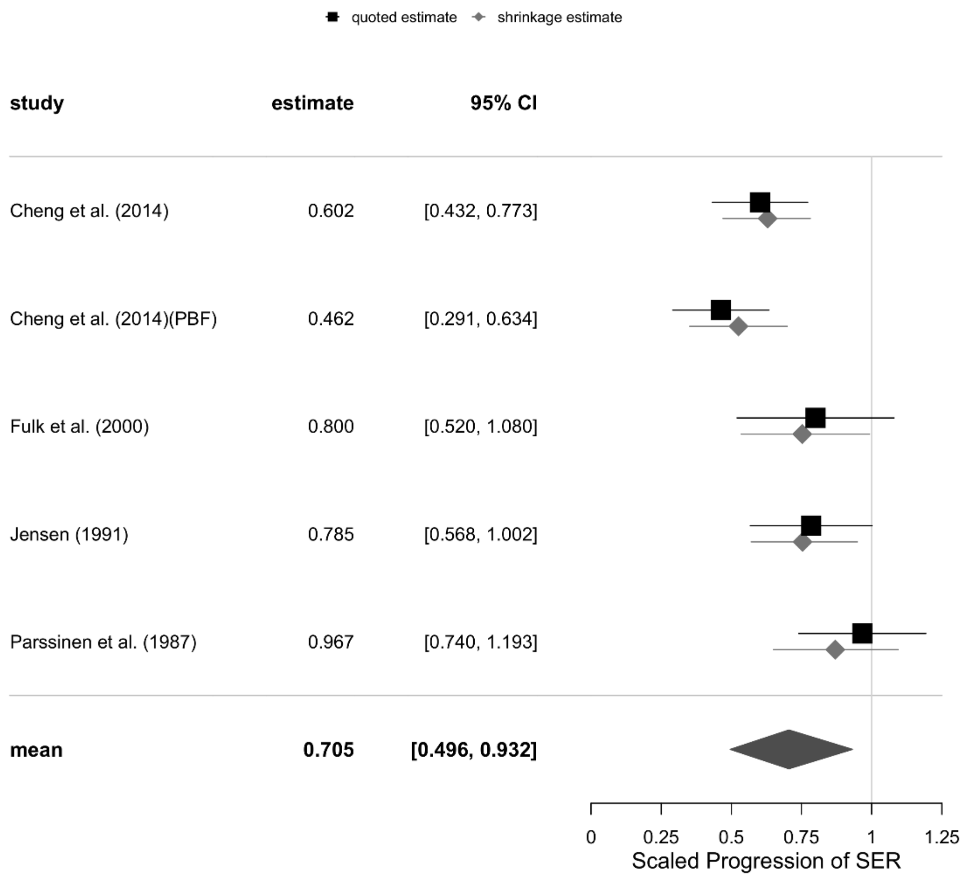

Figure S1. The forest plot of the Bayesian meta-analysis of the five RCTs using bifocal lenses to retard progression of SER after 12M follow-up. The first column of the forest plots references the source of the data, the second column gives the mean effect size obtained in the study expressed as a scaled progression, the third column gives the 95% credibility interval associated with the mean effect size. The last column shows the estimate of the mean effect size and the associated credibility intervals for the mean effect of each trial (black squares and lines) and the shrinkage estimates (grey diamonds and lines), which are corresponding estimates adjusted relative to μ and τ. The centre of the large diamond in the final row corresponds to the estimated mean effect size of all the trials included in the meta-analysis, and its width corresponds to the calculated 95% credibility interval.

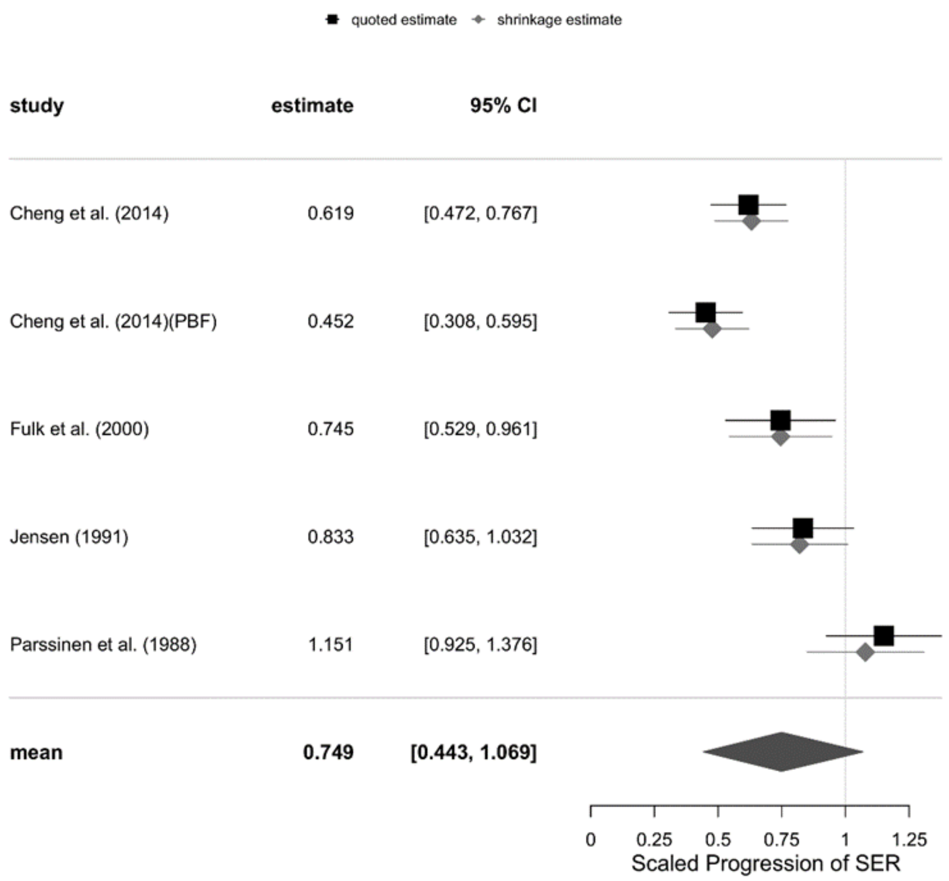

Figure S2. The forest plot of the Bayesian meta-analysis of the five RCTs using bifocal lenses to retard progression SER after 24M follow-up.

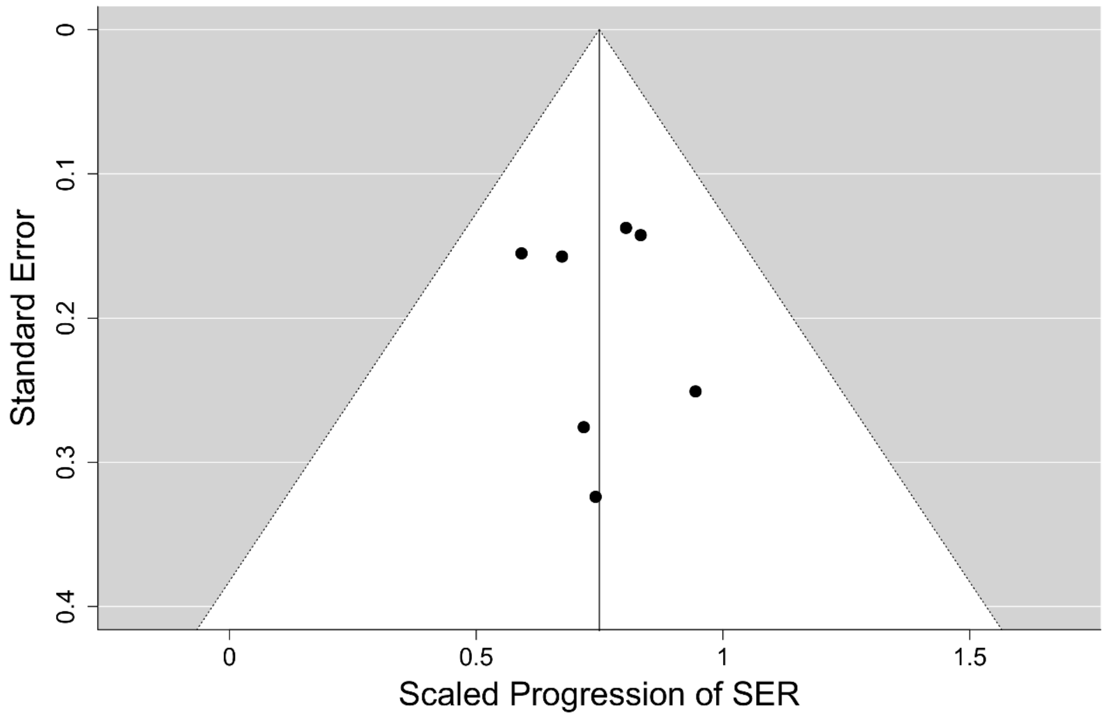

Figure S3: The funnel plot for the primary variable in PAL meta-analysis – the scaled progression of SER at the 6M follow-up with all trials included.

study	estimate	95% CI
Leung (1999) (A1.5D)	0.742	[0.107, 1.377]
Shih et al. (2001)	0.833	[0.554, 1.113]
Edwards et al. (2002)	0.674	[0.366, 0.982]
Hao et al. (2006)	0.718	[0.178, 1.258]
Hasebe et al. (2008)	0.592	[0.288, 0.896]
Yang et al. (2009)	0.944	[0.453, 1.436]
Hasebe et al. (2014)(A1.5D)	0.804	[0.534, 1.073]
**mean**	**0.732**	**[0.616, 0.848]**

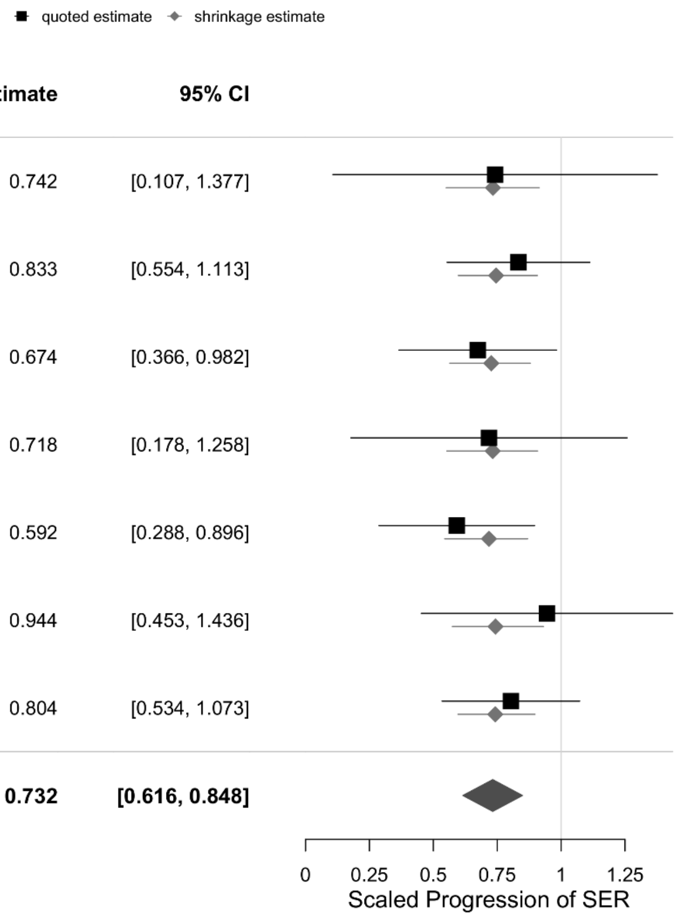

Figure S4: PAL versus SVL. Forest plot for the scaled progression in SER at the 6M follow-up with all the trials included.

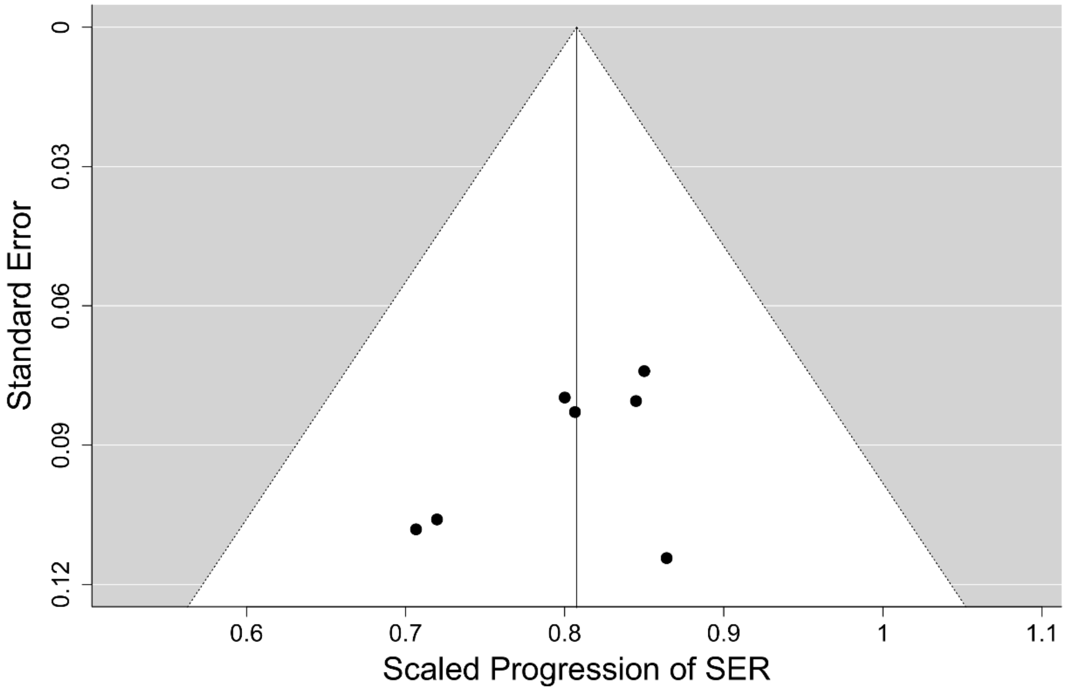

Figure S5: The funnel plot for the primary variable in PAL meta-analysis – the scaled progression of SER at the 18M follow-up with all trials included.

study	estimate	95% CI
Leung (1999) (A1.5D)	0.707	[0.495, 0.918]
Edwards (2002)	0.806	[0.644, 0.969]
Hao et al. (2006)	0.864	[0.640, 1.088]
Hasebe et al. (2008)	0.720	[0.512, 0.927]
Yang et al. (2009)	0.845	[0.687, 1.003]
Shih et al. (2011)	0.850	[0.705, 0.995]
Hasebe et al. (2014) (A1.50 D)	0.800	[0.644, 0.956]
**mean**	**0.803**	**[0.728, 0.877]**

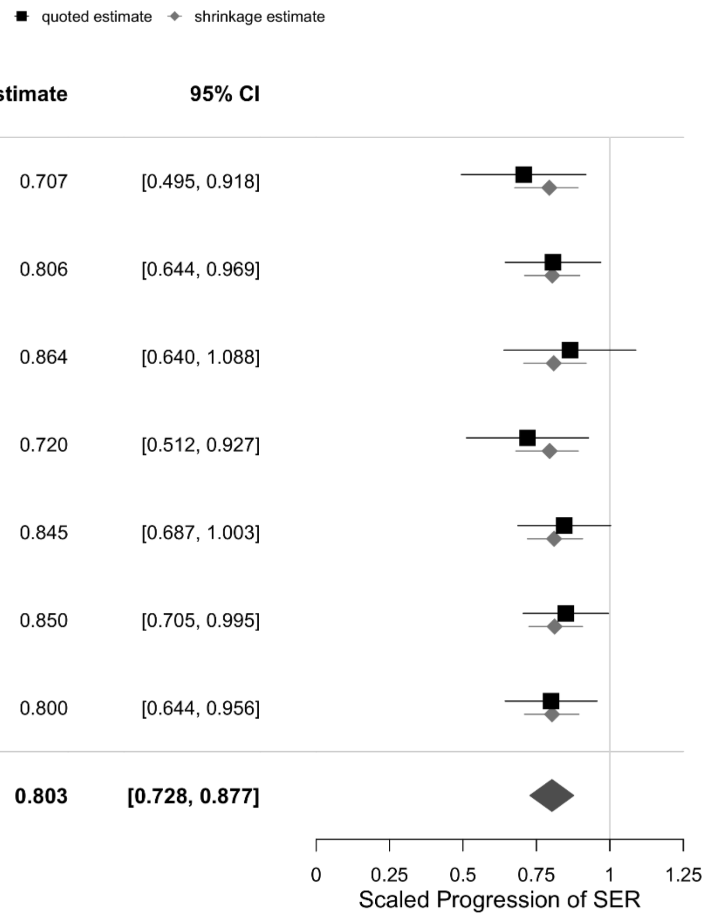

Figure S6: PAL versus SVL. Forest plot for the scaled progression in SER at the 18M follow-up with all the trials included.

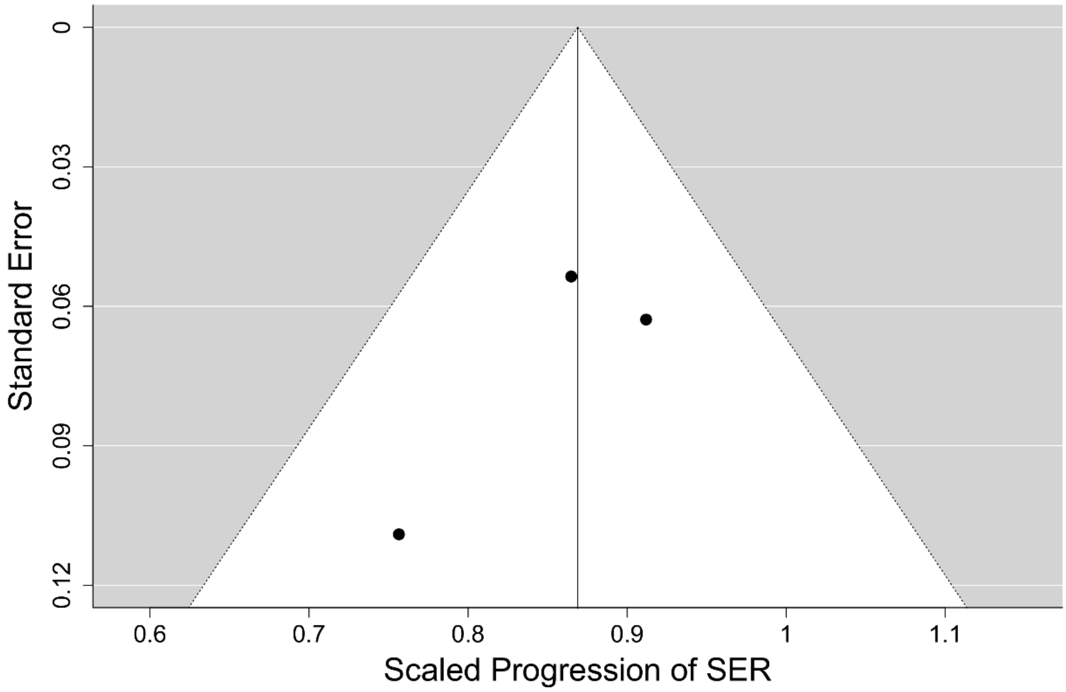

Figure S7: The funnel plot for the primary variable in PAL meta-analysis – the scaled progression of SER at the 36M follow-up with all trials included.

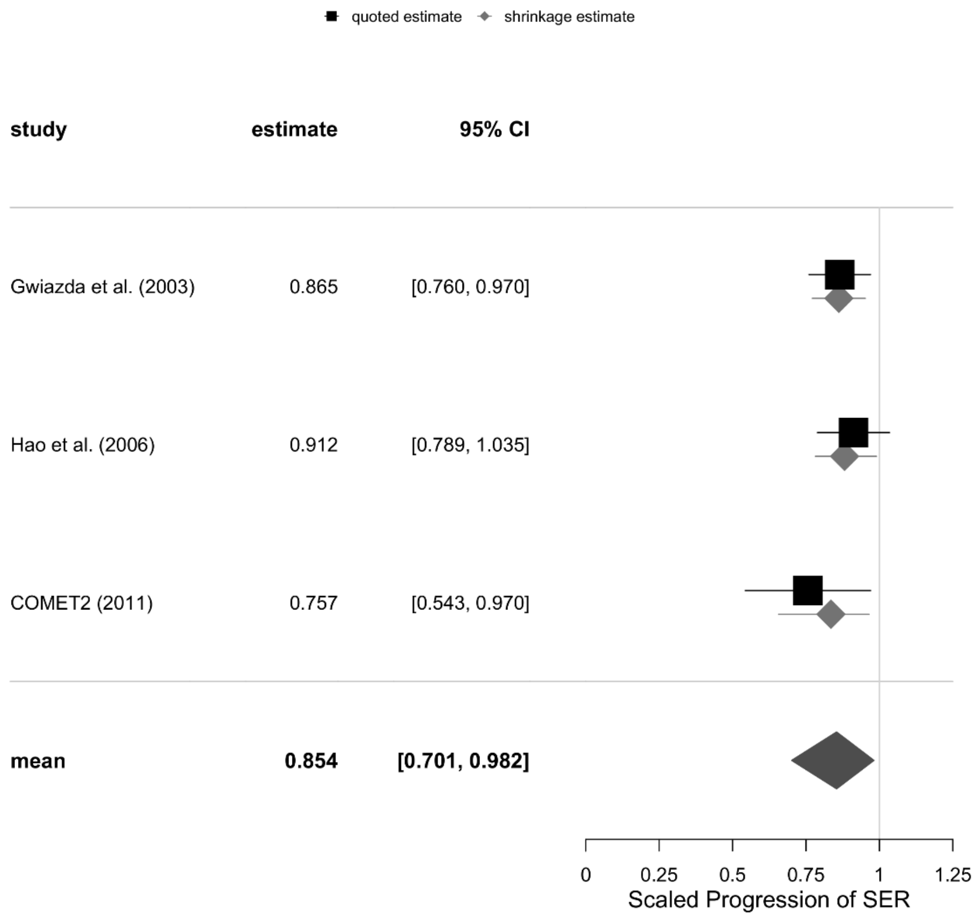

Figure S8: PAL versus SVL. Forest plot for the scaled progression in SER at the 36M follow-up with all the trials included.

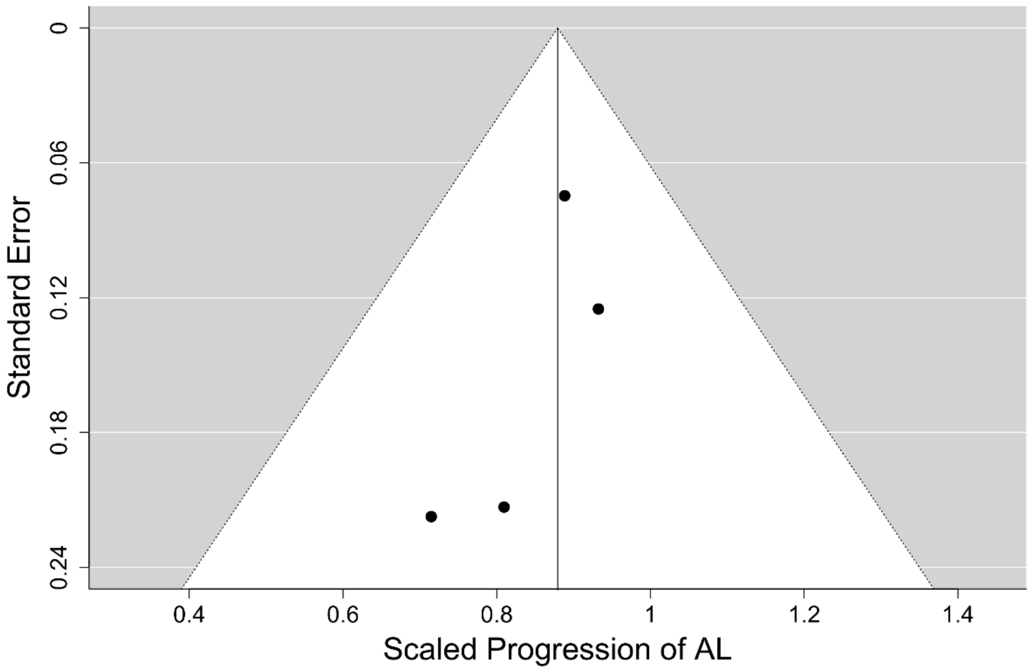

Figure S9: The funnel plot for the secondary variable in PAL meta-analysis – the scaled progression of AL at the 6M follow-up with all trials included.

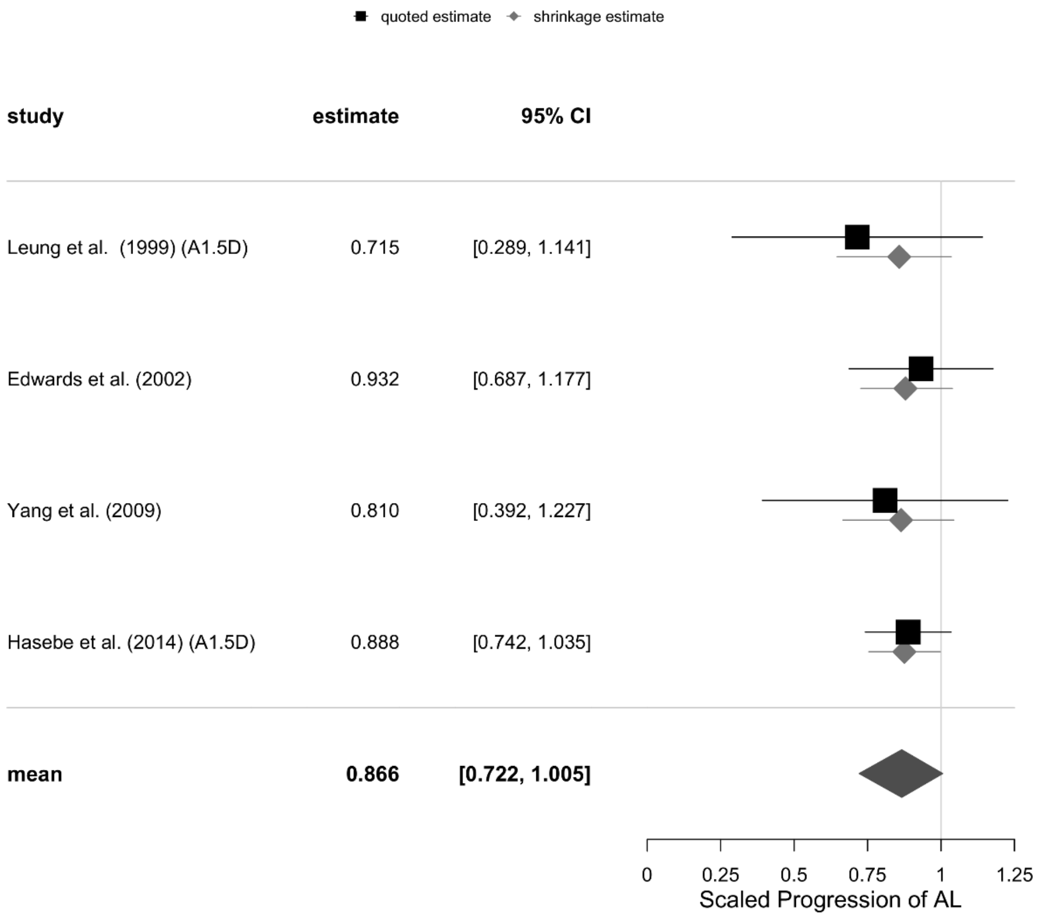

Figure S10: PAL versus SVL. Forest plot for the scaled progression in AL at the 6M follow-up with all the trials included.

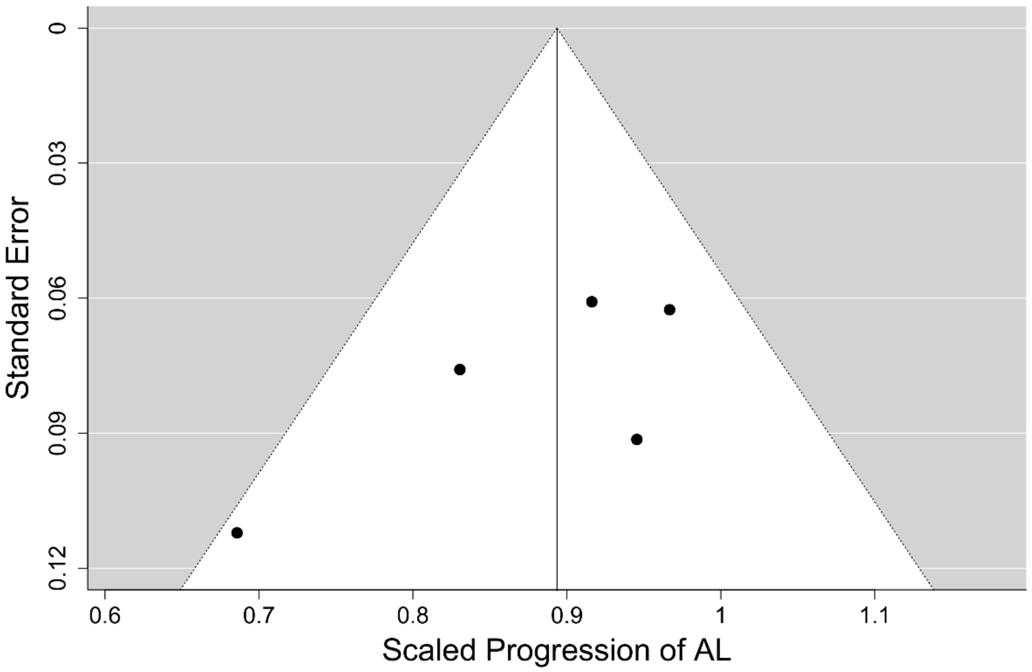

Figure S11: The funnel plot for the secondary variable in PAL meta-analysis – the scaled progression of AL at the 18M follow-up with all trials included.

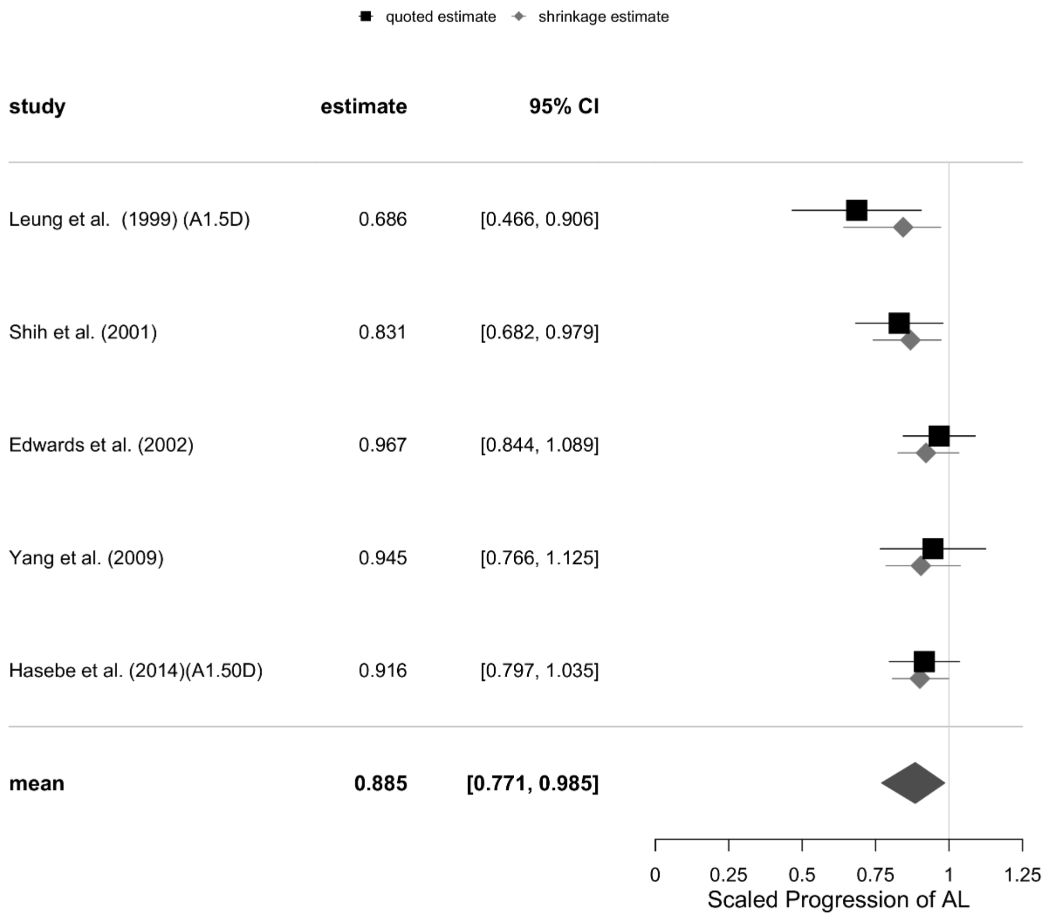

Figure S12: PAL versus SVL. Forest plot for the scaled progression in AL at the 18M follow-up with all the trials included.
